# Sensitivity and Specificity of Computed Tomography in the Evaluation of Bone Mineral Density in Mexican Patients with Breast Cancer

**DOI:** 10.7759/cureus.5505

**Published:** 2019-08-28

**Authors:** Ana Amador Martínez, Eleazar Lara Padilla, Juan Antonio Pérez Rodríguez, Alfonso Alfaro, Dania Guadalupe Solis Cano, Cindy Bandala, Nancy Guzman

**Affiliations:** 1 Radiology, Centro Médico ABC, CDMX, MEX; 2 Medicine, Instituto Politécnico Nacional, CDMX, MEX; 3 Neuroscience, National Rehabilitation Institute, CDMX, MEX

**Keywords:** bone mineral density, breast cancer, hounsfield units

## Abstract

Introduction

Breast cancer (BC) is the most frequently reported cancer among women - reported in 2012 as 25% of all cancers. BC has been related to the increased life and activity of osteoclasts, conferring a higher risk for osteoporosis/osteopenia. This study aimed to determine a cut-off point in Hounsfield units (HU) as well as the sensitivity and specificity of computed axial tomography (CT) in the diagnosis of osteoporosis/osteopenia in Mexican women with BC.

Material and methods

We included 108 patients with a histopathological diagnosis of BC treated at the ABC Medical Center in Mexico City. All patients were subjected to both dual X-ray densitometry and CT. The receiver operating characteristic (ROC) curve was used to identify the cutoff point and sensitivity and specificity were calculated, as were confidence intervals for the diagnoses of osteoporosis/osteopenia.

Results

The mean age was 58.49 ± 11.01 years. The cutoff point with the highest sensitivity (82%) and specificity (68%) was <157 HU for osteoporosis/osteopenia in patients with BC.

Conclusions

Women with BC are exposed to several risk factors for osteoporosis/osteopenia. The CT obtained for the general evaluation of these patients can also be used to evaluate bone mineral density, avoiding additional examinations and exposure to radiation, as well as the cost it confers, offering an earlier diagnosis of osteoporosis/osteopenia for its control.

## Introduction

Breast cancer (BC) is the second most common cancer in women [1,2]. Incidence of BC increases with age; it has been reported that over 75% of BC occurs in postmenopausal women [3]. Different studies show a connection between bone mineral density and BC [4,5]. The numerous factors favoring the appearance of osteoporosis in cancer patients are related to the tumor itself and to the antitumor treatment [6]. Chemotherapy-induced ovarian failure is a common cause of bone degradation among patients with breast cancer [7].

The recognition of osteoporosis as one of the late sequelae that may appear after the cure of cancer requires an early diagnosis of this disease in patients with an oncological background to adopt the appropriate preventive measures [8,9]. Bone mineral density is inversely proportional to the risk of fracture and is currently the best predictor to evaluate this parameter [10-14]. The risk of fracture for a patient with osteoporosis is 40%, with the most frequent sites of this event being the spine, the hip, and the wrist, among others [15]. In addition to conventional radiography, there are other imaging techniques such as dual X-ray absorptiometry (DXA) and computed tomography quantification, which has been developed to evaluate bone mineral content [16]. The International Society for Clinical Densitometry (ISCD) recommends that the BMD (Bone Mineral Density) test should be carried out on all women 65 years of age or older, as well as all men over 70 years of age [16]. On the other hand, the Hounsfield Units (HU) represent the relative density of body tissues according to a calibrated level scale, based on values for air (-1000 HU), water (0 HU), and bone density (+1000 HU) [17]. Literature indicates that bone quality can also be determined through the measurements of HU, which can be calculated from a region of interest using most modern radiology programs without additional costs or radiation [18]. A sensitivity of 86% and a specificity of 94% have been described for computed axial tomography (CT) in the diagnosis of osteoporosis [18]. DXA is currently the gold standard for assessing the bone mineral density and has been correlated with fracture risk. Although it is useful for evaluating osteopenia or osteoporosis, it poses certain methodological limitations [19]. Both DXA and CT can be used for the quantification of bone mineral density. Recently, several studies have proposed a potential correlation between BMD and the result of CT. There are different conclusions regarding the usefulness of CT to evaluate BMD: the majority suggests that CT provides accurate and reliable results in this tenor. CT has been suggested to have the potential to be considered an alternative method of timely detection of osteoporosis, without radiation or additional cost since CT can be analyzed for different aspects related to the patients' pathology [20-23]. Our study is a pioneer in showing the result of a CT scan as a diagnostic test for osteoporosis/osteopenia in Mexican patients with BC.

## Materials and methods

A total of 108 patients diagnosed with BC (clinical and histopathological diagnosis) - with densitometry and computed tomography studies using multidetector helical equipment, cross-sections were made with abdomen computed tomography protocol, detector coverage (mm) 40.0, helical thickness (mm) 2.5, pitch and speed (mm/rot) 1.375: 1, 55.00, rotation time (s) 0.6, mA 520, KV 120, with an approximate total dose report per patient CTD vol 11.14 (mGy), DLP 578.93 (mGy-cm) conducted at the ABC Cancer Center, Mexico City - were included in this study. Exclusions comprised patients with hypothyroidism, with treatments based on steroid corticosteroids, and with incomplete clinical records; unreliable DXA or CT results as patients with metallic material in vertebral bodies were also eliminated. Medical records were collected using the clinical file with prior approval from ABC Medical Center's ethics committee (folio TABC-17-22). Baseline characteristics were collected from the clinical history, including data on risk and comorbidity as well as hereditary family history, smoking habits, and excess weight or obesity by body mass index. The CT scan results were collected in HU from the evaluation of the vertebral body of L3 (trabecula) using PACS. A DXA analysis was performed by an observer-blinded from the DXA results on the lumbar spine and both coxofemoral joints. Results were classified according to the World Health Organization in standard deviations (SD), according to the parameter for age in our population (Osteopenia: -1 to -2.5 SD. Osteoporosis: -2.5 SD. Normal: up to -1 SD).

The densitometer's brand was Hologic, model Discovery WI with serial number 87039. The tomography equipment employed was a 64-slice GE CT scanner, lightspeed VCT model with serial number 5230VCTABC.

Means, standard deviations, frequencies, and percentages were determined. The diagnostic test parameters were determined for qualitative data as well as the receiver operating characteristic (ROC) curve to obtain a cut-off point in HU for osteoporosis/osteopenia. Kolmogorov-Smirnov, chi-square, ANOVA and the Pearson and Spearman correlation tests were applied. The data analysis was performed with the program SPSS v19 and Epidat 3.1. A confidence interval of 95% was taken for all tests and determinations.

## Results

A total of 108 patients diagnosed with BC were studied. The average age was 58.49 ± 11.01 years, with a 48 years range (34 to 82 years). The frequencies of risk factors related to lifestyle and comorbidities were as follows: 29.6% of the patients were overweight and 16.7% were obese. The minimum BMI was 18 kg/m^2^ and the maximum was 39 kg/m^2^. Regarding the gynecological-obstetric history, the frequency of early menarche was 3.7% (four cases), none of the patients had late menopause and only 4.6% (five cases) was nulliparous. Stage III-IV had a frequency of 22.2% (24 cases), metastasis at the time of diagnosis was 20.4% (22 cases). Women who were in stage III-IV had 18 times more risk (95% confidence interval of 5.83-56.14); that is, metastasis was related in 68.2% to stage III-IV (p = 0.0001). Only 9.3% (10 cases) were treated with hormone therapy and 37.4% (40 cases) with surgery. Regarding the immunohistochemical markers, 48.1% of cases had triple-negative profile (Negative estrogen receptor/negative progesterone receptor/negative HER2).

The bone density evaluated by DXA showed that 55.6% of the patients presented osteoporosis/osteopenia (45.4% Osteopenia, 10.2% Osteoporosis). The DXA result was related to the risk age for menopause and stage III-IV (Table 1).

**Table 1:** DXA result related to the risk age for menopause and stage III-IV. *Statistical significance, P-value <0.05, Chi square DXA: Dual X-ray absorptiometry

	DXA Study Results						
	Normal (n = 38)	Osteopenia (n = 56)	Osteoporosis (n = 14)	P -value	
Age >48 years	38% (32)	50% (42)	12% (10)	0.04*	
Stage III-IV	25% (6)	58.3% (14)	16.7% (4)	0.03*	

Table 2 shows the HU global averages obtained through CT in relation to the qualitative result of the DXA (normal, osteopenia, osteoporosis). This same table also shows the mean HU values obtained through the CT scan in the risk age for menopause and relation to the categorical result of the DXA (normal, osteopenia, osteoporosis).

**Table 2:** Bone density assessed by CT (HU) in relation to DXA results with the age of risk for menopause. *Statistical significance SD: Standard deviation; CT: Computed axial tomography; DXA: Dual X-ray absorptiometry.

		Age of Risk for Menopause	
DXA results	Global Mean ± SD	>48 years Mean ± SD	<48 years Mean ± SD
Normal	187.84 ± 64.4	166.03 ± 56.71	231.46 ± 59.4
Osteopenia	139.19 ± 40.1	134.39 ± 38.3	164.42 ± 44.6
Osteoporosis	93.73 ± 21.6	95.74 ± 21.73	73.63
P value	0.0001*	0.0001*	0.007*

**Figure 1:**
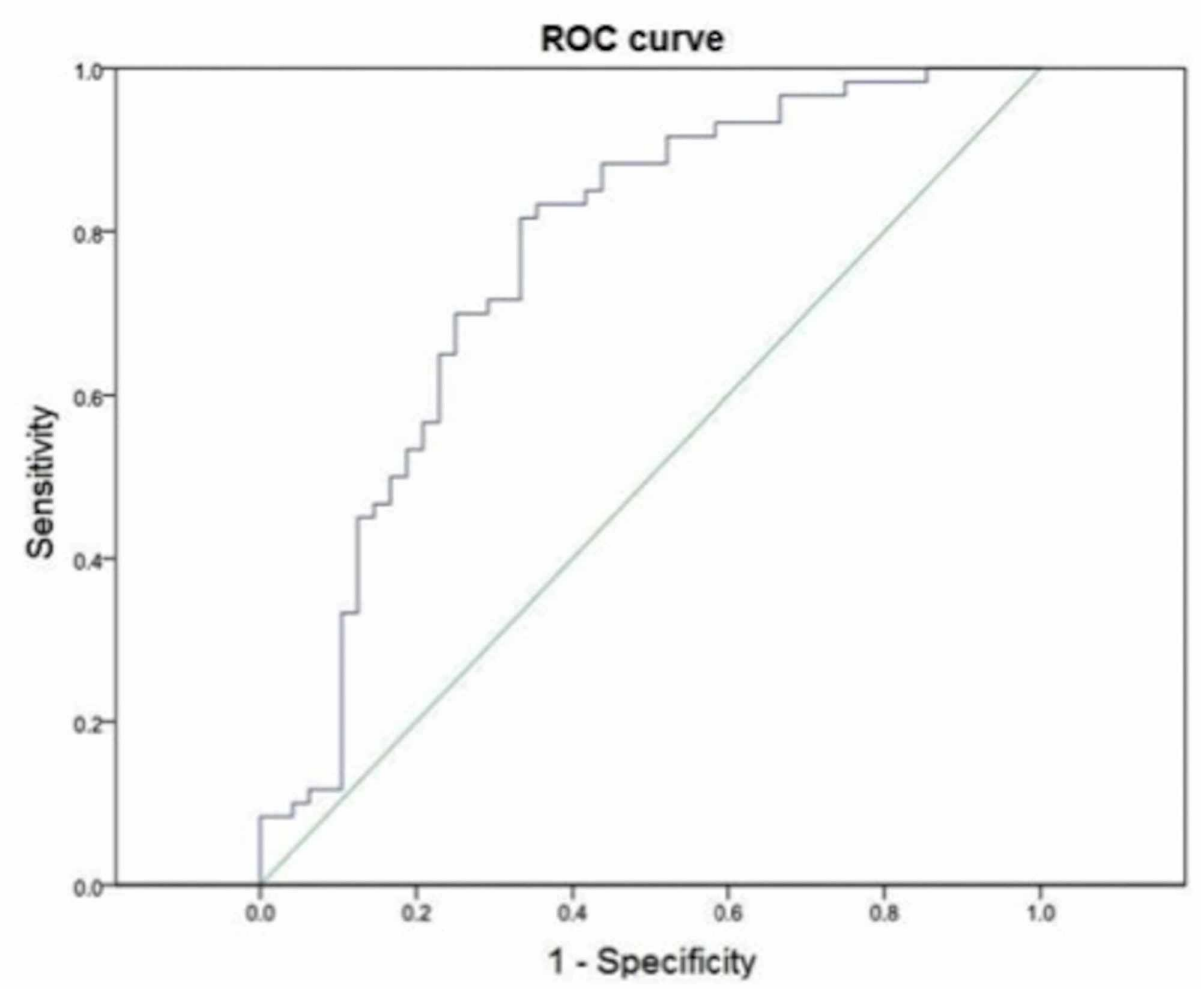
ROC curve of the CT study (HU) to evaluate bone mineral density (BMD) in patients diagnosed with BC. ROC: Receiver operating characteristic; BC: Breast cancer.

Figure 1 shows the ROC curve, where the maximum point of sensitivity and specificity for CT (HU) is shown in relation to the result of osteoporosis/osteopenia according to DXA. The optimal cut-off point was <157 HU for osteoporosis/osteopenia in patients with BC, reaching a sensitivity of 82% and a specificity of 68%, with an area under the curve of 0.76 (p = 0.0001) and a confidence interval of 95% from 0.67 to 0.86.

A contingency table was made between the diagnosis of osteoporosis/osteopenia (cut-off point of <157 HU) evaluated by CT and by DXA. Table 3 shows 35 cases of true positives between both diagnostic methods.

**Table 3:** CT scan results with a cut-off point of 157 HU in relation to the DXA results in patients with BC. CT: Computed axial tomography; DXA: Dual X-ray absorptiometry; BC: Breast cancer.

			DXA		Total (n = 108)
			Osteoporosis/osteopenia (N = 60)	Normal (n = 48)	
CT	<157 HU	Osteoporosis/osteopenia	58.3% (35)	64.6% (31)	61.1% (66)
	>157 HU	Normal	41.7% (25)	35.4% (17)	38.9% (42)

Table 4 shows the CT scan's diagnostic test parameters, employing the cutoff point of 157 HU, and the gold standard (DXA characterized in healthy patients).

**Table 4:** Diagnostic test parameters for CT scan with a 157 HU cut-off point and DXA results in patients with BC. CT: Computed axial tomography; DXA: Dual X-ray absorptiometry; BC: Breast cancer; PPV: Positive prognostic value; NPV: Negative prognostic value.

	Sensitivity (CI 95%)	Specificity (CI 95%)	PPV (CI 95%)	NPV (CI 95%)	Prevalence (CI 95%)
CT	58.33% (45.03 to 71.64)	35.42% (20.84 to 49.99)	53.03% (40.23 to 65.83)	40.48% (24.44 to 56.51)	55.56% (45.72 to 65.39)

Finally, we observed that the HU obtained by CT scan negatively correlated with age (r = -0.53, p = 0.0001) and the DXA result (r = -0.53, p = 0.0001).

## Discussion

The average age our patients presented at the time of diagnosis of BC was similar to that previously reported for the Mexican population [24-27]. Regarding excess weight and obesity, in our study, we observed lower frequencies concerning other studies conducted in our country [28]. The prevalence of osteoporosis found in our population was similar to that described in studies of osteoporosis in Europe: it was only 1.8% lower than that described in the European population [28]. According to the findings in the DXA values, the patients with osteoporosis were mostly menopausal women; when comparing clinical stages, those located in stage III-IV had the highest percentage of osteoporosis (36.4%).

Regarding the results obtained by CT pre-menopausal patients with normal DXA presented 65.43 less HU than those found in menopausal patients with normal DXA, as expected. Likewise, when comparing the results in patients on clinical stages III-IV but with a normal DXA, these were higher (86.4 more) than that obtained in abnormal DXA patients but with early clinical stages (I-II-III). This can presumably be due to the presence of metastatic lesions as well as the biochemical processes of the activation of beta adrenergic receptors [29]. Through the CT scan, a cut-off level for osteoporosis/osteopenia equal to or <157 HU was obtained with a sensitivity of 82%, which is very similar to that reported by Pickhardt et al. in 2013. They reported a 90% sensitivity for a cut-off level of 160 HU, even though their study was of the North American population and included both sexes [21]. Other studies exist where authors correlate DXA with CT: Batawil and Sabiq in 2012 described a cut-off level of 203 HU to exclude osteopenia/osteoporosis [30]. This may be because this study was conducted in the country of Saudi Arabia, with a group of women with various previous diagnoses and only some of them with BC.

According to the results from this clinical investigation, values were obtained using CT with Hounsfield units (HU) for osteoporosis/osteopenia and normal bone density. The mean for patients with BC with a normal DXA was 187.84 HU; it was 139.19 HU for patients with a DXA showing osteopenia, and 93.73 HU for patients with a DXA for osteoporosis. This difference in HU values was both statistically significant and similar to other studies. We conclude that computed tomography could provide a bone density profile employing Hounsfield units in all patients who undergo this study for other medical reasons. It is relevant for the clinician since the state of bone metabolism should not be forgotten in the evaluation of the patient with breast cancer, the timely detection will allow him to reduce fracture risks.

Mexican patients with breast cancer are a good example: by taking advantage of the studies that are practiced for follow-up and/or approach during clinical staging, the time-cost-radiation of dual X-ray absorptiometry can be avoided. The optimal cut-off point for osteoporosis/osteopenia through CT was equal to or less than 157 HU, reaching a sensitivity of 82% and a 68% specificity, with a 95% CI. The resulting HU correlated with age: pre-menopausal patients showed an average of 231.46 HU, unlike patients with BC and menopausal women who had an average of 166.03 HU. These results show that normal bone density parameters should be established in cancer patients with both DXA and CT studies. Normal bone density parameters should also be reestablished in the gold standard that is densitometry when it is determined in oncological patients.

Limitations

One limitation of our study was that it was not possible for us to validate our results, we only reported the descriptive findings and we proposed a cut-off point, for our population but we need to perform further studies.

## Conclusions

According to the results obtained by CT in our clinical research, the average for breast cancer patients with normal densitometry was 187.84 HU, for patients with osteopenia in densitometry was 139.19 HU and 93.73 HU for osteoporotic patients by densitometry; this difference in values of HU was statistically significant. The optimal cut-off point obtained by ROC curve for osteoporosis/osteopenia by CT was equal or less than 157 HU, reaching a sensitivity of 82% and 68% specificity with a 95% CI. Therefore, we conclude that computed tomography can provide a bone density profile using the Hounsfield units.
